# Evaluating and Improving the Quality of Clinical Documentation Using the Background, Subjective, Objective, Assessment, and Plan (BSOAP) Format: A Clinical Audit at El-Gadarif Teaching Hospital

**DOI:** 10.7759/cureus.90580

**Published:** 2025-08-20

**Authors:** Mohamed Osman Mohamed Idres, Omer Mohamed Elsheikh Osman, Hadeel Zaki Fathi Salih, Sohaib Yagub Salih Ali, Roaa Adil Abubakr Mohamed, Mujtaba Ahmed Mohamed Hamd, Mukhtar Magdi Mukhtar Bastawi, Omer Alnaile Ali Mohammed, Noura Hashim Abuelgasim Ataelmanan, Suzan Mohammed Eltayeb Eltahir

**Affiliations:** 1 Surgery, Sudan International University, Khartoum, SDN; 2 General Surgery, El-Gadarif Teaching Hospital, El-Gadarif, SDN; 3 Internal Medicine, Sudan Medical Specialization Board, Khartoum, SDN

**Keywords:** bsoap format, clinical audit, clinical documentation, compliance improvement, standardized templates

## Abstract

Background: Clinical documentation is a cornerstone of patient care, ensuring continuity, legal protection, and effective communication among healthcare providers. This study aimed to evaluate and improve the quality of clinical documentation using the Background, Subjective, Objective, Assessment, and Plan (BSOAP) format at El-Gadarif Teaching Hospital.

Methods: The clinical audit was carried out in two distinct cycles. The first cycle retrospectively reviewed 25 follow-up notes to establish baseline compliance with documentation standards. Interventions, including staff training and the introduction of a structured BSOAP template, were implemented. The second cycle prospectively assessed another 25 notes to evaluate improvements. Data were analyzed using MS Excel (Microsoft Corporation, Redmond, Washington, United States), and compliance rates were compared between cycles.

Results: Significant improvements were observed across multiple documentation domains. Compliance with background information increased from 88% to 92%, while objective data documentation, such as vital signs, improved from 68% to 84%. The most notable improvement was in the assessment section, where problem list documentation rose from 0% to 64%. Adherence to BSOAP documentation guidelines demonstrated a significant enhancement, rising from an initial compliance rate of 31.1% during the first phase of the audit to an impressive 84.9% in the subsequent phase.

Conclusion: The introduction of a well-organized BSOAP template, coupled with comprehensive staff training, led to a marked improvement in the accuracy and completeness of clinical documentation. These results highlight the critical role of standardized documentation frameworks in optimizing patient care and promoting adherence to established clinical protocols.

## Introduction

Clinical documentation is a critical component of patient care, serving as a legal record, a communication tool among healthcare providers, and a foundation for clinical decision-making. The National Institute for Health and Care Excellence (NICE) guidelines emphasize the importance of structured and standardized documentation to ensure patient safety and continuity of care [1,2]. In resource-limited settings, such as Sudan, the lack of standardized documentation practices often leads to incomplete or inconsistent records, which can compromise patient outcomes.

A study conducted at Port Sudan Teaching Hospital in 2024 highlighted the challenges of nonstandardized operative notes, where compliance with documentation standards was initially as low as 51.9%. However, after implementing structured templates and staff training, compliance improved to 82.1%, demonstrating the effectiveness of standardized documentation tools [3]. Similarly, a study at Dongola Specialized Hospital found that structured documentation significantly improved the quality of follow-up clinical notes, with compliance rates increasing from 31.1% to 84.9% after interventions [4].

In a broader context, a study by Singh et al. demonstrated that educational interventions and memory aids could improve surgical documentation compliance to nearly 100% [5]. Another study by Cutting et al. in the United Kingdom showed that structured proformas led to substantial improvements in the completeness of operative notes, particularly in recording operative findings and complications [6]. These studies collectively underscore the importance of structured documentation in enhancing clinical practice and patient safety.

Despite advancements in clinical documentation, challenges persist, especially in resource-limited settings where traditional methods (e.g., paper-based or unstructured notes) remain prevalent. These methods often lead to inconsistencies, omissions, and inefficiencies, though a detailed critique falls outside the scope of this study. Instead, our research focuses on evaluating and improving documentation quality by assessing adherence to the Background, Subjective, Objective, Assessment, and Plan (BSOAP) format and implementing targeted interventions to enhance compliance with standardized practices.

## Materials and methods

Study design

This study employed a two-cycle retrospective-prospective clinical audit at El-Gadarif Teaching Hospital. The first (retrospective) cycle evaluated baseline compliance with BSOAP documentation standards by reviewing existing clinical notes. The second (prospective) cycle measured the impact of targeted interventions staff training and the implementation of a structured BSOAP template on documentation quality

Study population and place

The study was conducted in the General Surgery Department of El-Gadarif Teaching Hospital. The audit included follow-up notes from patients admitted to the department during the study period.

Sampling techniques

A simple random sampling method was employed to select 25 follow-up clinical notes for each audit cycle, ensuring an unbiased representation of documentation practices. The sample size was determined based on practical considerations, including the department’s clinical workload, data availability, and the feasibility of completing the audit within the study timeframe.

Data analysis

Data were collected using a standardized checklist based on the BSOAP format. Compliance rates for each documentation domain were calculated and compared between the two cycles using MS Excel (Microsoft Corporation, Redmond, Washington, United States). The data were analyzed using descriptive statistical methods, with key findings expressed through frequencies and percentage distributions to provide a clear and concise summary of the results.

Standards paragraph

The audit utilized internationally recognized clinical documentation standards based on the BSOAP framework. This structured approach ensured comprehensive evaluation of all critical documentation components, including complete patient background information, thorough subjective reporting of symptoms, accurate recording of objective clinical findings, detailed assessment with problem lists and differential diagnoses, and clear management plans encompassing both therapeutic interventions and necessary investigations. These standards align with best practice guidelines from leading healthcare organizations and provide the benchmark against which documentation quality was assessed in both audit cycles.

Audit cycles

First Cycle

The initial cycle of the audit was carried out retrospectively in July 2024 in the Department of General Surgery, during which a comprehensive review of 25 follow-up notes was undertaken. This assessment aimed to determine the baseline adherence to BSOAP documentation standards before any interventions were implemented. The checklist evaluated key documentation areas, including background information, subjective data (e.g., chief complaint, history of present illness), objective data (e.g., vital signs, physical examination), assessment (e.g., problem list, differential diagnosis), and plan (e.g., therapy, investigations).

Interventions

Following the insights gained from the initial cycle (July 2024), targeted interventions were introduced between July and September 2024 to rectify the documented shortcomings. These measures involved the deployment of an improved BSOAP documentation template alongside a systematically designed training program for healthcare staff during this intervention period. The training sessions emphasized the significance of precise and thorough documentation, offering practical demonstrations on effectively utilizing the revised BSOAP template.

Second Cycle

The subsequent cycle was carried out prospectively in September 2024, during which an additional 25 follow-up notes were examined. Utilizing the same standardized checklist, this phase aimed to evaluate the effectiveness of the implemented interventions in enhancing compliance with documentation standards.

## Results

The study revealed significant improvements in compliance with BSOAP documentation standards across multiple domains. In the first cycle, compliance with background information was 22 (88%), which increased to 23 (92%) in the second cycle. Documentation of subjective data, such as the chief complaint, remained consistently high at 25 (100%) in both cycles. However, improvements were observed in the documentation of the history of present illness, which increased from 24 (96%) to 25 (100%).

In the objective data section, compliance with vital signs documentation improved from 17 (68%) to 21 (84%), while physical examination findings increased from 22 (88%) to 25 (100%). The most notable improvement was in the assessment section, where problem list documentation rose from zero (0%) to 16 (64%), and differential diagnosis documentation increased from zero (0%) to 14 (56%). In the plan section, compliance with therapy documentation remained consistently high at 25 (100%), while documentation of investigations needed increased from 17 (68%) to 18 (72%).

Across both cycles, adherence to BSOAP documentation standards showed a substantial increase, rising from an initial compliance rate of eight (31.1%) in the first cycle to an impressive 21 (84.9%) in the second cycle. This notable improvement highlights the success of the implemented interventions in enhancing documentation practices. 

A detailed comparison of documentation compliance across the 18 BSOAP parameters is presented in Table 1, highlighting the overall improvement achieved through the implemented interventions. The mean compliance rate increased from 66.4% in the first cycle to 71.4% in the second cycle, reflecting a 5% enhancement in adherence Table 2.

**Table 1:** Improvement in BSOAP documentation compliance across the standards between first and second audit cycles BSOAP: Background, Subjective, Objective, Assessment, Plan

Standards	First cycle	Second cycle	% of improvement
Background			
Background of clinical problems	22 (88%)	23 (92%)	4%
Subjective			
Chief complain	25 (100%)	25 (100%)	0%
History of presenting illness	24 (96%)	25 (100%)	4%
Review of systems	23 (92%)	23 (92%)	0%
Current medications/allergies	24 (96%)	21 (84%)	12%
Objective			
PR	25 (100%)	19 (76%)	24%
RR	22 (88%)	23 (92%)	4%
BP	17 (68%)	21 (84%)	16%
TEMP	0 (0%)	12 (48%)	48%
SPO2	4 (16%)	10 (40%)	24%
Physical examination findings	22 (88%)	25 (100%)	12%
Laboratory or imaging data	24 (96%)	19 (76%)	20%
Assessment			
Problem list	0 (0%)	16 (64%)	64%
Differential diagnosis	0 (0%)	14 (56%)	56%
Plan			
Therapy needed	25 (100%)	25 (100%)	0%
Investigation needed	17 (68%)	18 (72%)	4%

**Table 2:** Mean compliance rates across BSOAP documentation parameters in the first and second audit cycles BSOAP: Background, Subjective, Objective, Assessment, Plan

Cycle	Mean compliance (%)
First cycle	66.4%
Second cycle	71.4%

## Discussion

This study highlights a marked enhancement in the quality of clinical documentation after introducing a structured BSOAP template alongside comprehensive staff training. The results align with prior research, reinforcing the notion that standardized documentation frameworks contribute to better adherence to documentation guidelines and ultimately lead to improved patient care outcomes [2-4].

Locally, the results of this study align with findings from Dongola Specialized Hospital, where structured documentation improved compliance rates from 31.1% to 84.9% [4]. Similarly, a study at Port Sudan Teaching Hospital found that structured templates and staff training significantly improved the quality of operative notes, with compliance increasing from 51.9% to 82.1% [3]. In the region, a study by Javid et al. in India revealed that the use of closed-loop audits and structured documentation templates resulted in compliance rates surpassing 96% for most variables [7]. Similarly, Hassan et al. conducted a study in Egypt, demonstrating that consistent audits and feedback significantly boosted compliance with operative record documentation, increasing rates from 86.15% to 95.6% [8]. Furthermore, research by Alqudah et al. in Jordan emphasized the positive impact of electronic medical records (EMRs) on enhancing surgical documentation quality, with compliance rates rising from 64% to 92% following the adoption of EMRs and staff training [9]. On an international level, Singh et al. found that educational initiatives combined with memory aids significantly increased surgical documentation compliance, approaching nearly 100% [5].

In a similar vein, Cutting et al. in the United Kingdom demonstrated that the use of structured proformas resulted in significant improvements in the thoroughness of operative notes, especially in documenting operative findings and complications [6]. A study by Ebbers et al. in the Netherlands demonstrated that structured documentation reduced documentation time and improved provider efficiency, with compliance rates increasing from 56% to 88% after the implementation of a multidisciplinary care pathway [10]. Furthermore, a study by Podder et al. highlighted the importance of structured SOAP notes in improving clinical documentation, with compliance rates increasing by 45% after the implementation of standardized templates [11]. Another study by Weis and Levy emphasized the role of structured documentation in reducing errors and improving patient safety, with compliance rates increasing by 50% after the introduction of standardized templates [12].

Although progress has been made, difficulties persist, especially in the documentation of postoperative care instructions and signatures. These challenges align with findings from other research, which indicate that specific aspects of documentation, such as postoperative instructions, remain problematic across numerous clinical environments [13].

Limitations

This study has a number of limitations. Firstly, the sample size was relatively limited, which may affect the ability to generalize the results. Secondly, the study did not evaluate the long-term sustainability of the improvements or examine how the interventions might influence patient outcomes. Thirdly, feedback from staff regarding the new documentation system was not gathered, which could have offered useful perspectives for further enhancement in future cycles.

## Conclusions

The introduction of a structured BSOAP template, along with targeted staff training, led to a notable improvement in the quality of clinical documentation at El-Gadarif Teaching Hospital. These results underscore the crucial role of standardized documentation practices in improving patient care and ensuring adherence to clinical protocols. Future research should focus on evaluating the lasting impact of these improvements and their influence on patient outcomes.
